# Ethosomal Nanocarriers for Trans-Resveratrol Delivery: Formulation, Physicochemical Characterization, Stability, and In Vitro Release Performance

**DOI:** 10.3390/pharmaceutics18101237

**Published:** 2026-09-29

**Authors:** Yasemin Yağan Uzuner, Hakan Sevinç

**Affiliations:** 1 Department of Pharmaceutical Technology, Faculty of Pharmacy, Acibadem Mehmet Ali Aydinlar University, 34752 Istanbul, Türkiye; 2 Department of Pharmaceutical Technology, Faculty of Pharmacy, Yeditepe University, 34752 Istanbul, Türkiye; 3 Biota Research & Development Center, 34785 Istanbul, Türkiye

**Keywords:** trans-resveratrol, ethosomes, antioxidant, photoaging, dermacosmetics

## Abstract

**Background:** Trans-resveratrol (3,5,4′-trihydroxystilbene) is a natural polyphenolic antioxidant widely used in anti-aging dermocosmetics for its strong radical-scavenging capacity and its activation of cell-protective pathways such as sirtuin 1 (SIRT1). However, its poor aqueous solubility, photochemical lability, and low bioavailability limit its incorporation into topical formulations and its delivery into the skin. **Objective:** In this study, ethosomal nanocarriers were designed as a phospholipid–ethanol vesicular system to solubilize, stabilize, and control the release of trans-resveratrol for dermocosmetic applications. Microfluidization is not a commonly used method; however, circulating the formulation through the interaction chamber under optimized pressure can produce ethosomes with desirable colloidal stability by this simple process. **Methods:** Resveratrol-loaded ethosomes were prepared with synthetic phosphatidylcholine (Lipoid P75), ethanol, and vitamin E. Microfluidization was optimized by varying the number of high-pressure homogenization cycles and the applied pressure. Vesicle size, size distribution and distribution uniformity, zeta potential, pH, conductivity, density, and long-term stability were monitored for up to 180 days; morphology was examined by cryogenic scanning electron microscopy (cryo-SEM) and molecular compatibility by Fourier-transform infrared (FTIR) spectroscopy. A trans-resveratrol high-performance liquid chromatography (HPLC) assay was developed and validated according to International Council for Harmonisation (ICH) Q2 guidelines for quantitative analysis. Encapsulation efficiency was determined by HPLC after ultracentrifugation, cytotoxicity was assessed in human keratinocytes (HaCaT), and in vitro release was evaluated using Franz diffusion cells with two different membranes. **Results:** All ethosome formulations yielded a nanoscale size distribution (median diameter around 190 nm for loaded and around 90 nm for unloaded) and good colloidal stability, with absolute zeta potentials above the 30 mV threshold at early time points and a skin-compatible pH (around 6.5). The optimized formulation (T16; 1.5% *w*/*w* trans-resveratrol, 5% *w*/*w* phosphatidylcholine (Lipoid P75), 0.3% *w*/*w* vitamin E and 30% *w*/*w* ethanol, processed with seven microfluidization cycles) achieved a high encapsulation efficiency (EE) of 95.5% on day 1. 84.2% EE was retained after 180 days, consistent with strong partitioning of the lipophilic active into the ethanol–phospholipid bilayer. FTIR confirmed preservation of the phospholipid bilayer and indicated non-covalent loading, with the resveratrol bands largely masked by the dominant lipid signals. Cryogenic Scanning Electron Microscopy (Cryo-SEM) confirmed near-spherical vesicles with narrow size distribution. In vitro release showed a sustained, controlled release profile relative to a 1.5% *w*/*w* resveratrol solution. Slower diffusion across the skin-mimicking Strat-M membrane was observed compared to cellulose acetate membrane. **Conclusions:** Optimized trans-resveratrol-loaded ethosomes represent a stable, efficient vesicular system enabling formulation stability and controlled topical release. The antioxidant and photoprotective efficacy of the loaded system was not assessed in this study and is identified as a topic for future work.

## 1. Introduction

Human skin ages through two overlapping processes. Intrinsic (chronological) aging is driven by time and genetically programmed changes, whereas extrinsic aging is dominated by environmental insults such as chronic ultraviolet (UV) exposure, a process called photoaging that accounts for most of visible symptoms of aging [1,2]. A central mechanism common to both ageing is oxidative stress: UV radiation and metabolic activity generate reactive oxygen species (ROS) that damage lipids, proteins, and Deoxyribonucleic Acid (DNA), and that activate the mitogen-activated protein kinase and Nuclear Factor kappa-light-chain-enhancer of activated B cells (NF-κB) signaling cascades. These cascades up-regulate matrix metalloproteinases (MMPs), which destroy dermal collagen and elastin, while at the same time down-regulating new collagen synthesis. These processes result in wrinkling, loss of firmness and elasticity, and dyspigmentation [2,3,4]. All these processes are increased in photoaging. Since ROS are the main reason for this cascade, topical antioxidants have become very important for anti-aging dermacosmetics [4].

Resveratrol (3,5,4′-trihydroxystilbene) is a stilbenoid polyphenol produced by many plants as a phytoalexin, which is a defensive compound synthesized in response to microbial infection, UV radiation, or physical injury [5]. It can be found in food mainly in grape skins and red wine, as well as in peanuts and berries. Its popular reputation is tied to the so-called “French paradox”: the epidemiological observation, first mentioned by Renaud and de Lorgeril, that populations in southern France have relatively low rates of coronary heart disease although their diet is rich in saturated fat. This effect is attributed in part to regular moderate-level consumption of polyphenol-rich red wine [6]. The molecule exists as two geometric isomers; the *trans*-isomer is the biologically dominant and more stable form and is the one intended in most therapeutic and cosmetic applications.

Resveratrol has the molecular formula C_14_H_12_O_3_ and a molar mass of 228.25 g/mol, and has three phenolic hydroxyl groups that cause much of its antioxidant, radical-scavenging properties. An important practical limitation is its solubility and stability profile: resveratrol is poorly soluble in water (0.03 g/L) but freely soluble in ethanol, is photolabile (the trans-isomer isomerizes to the cis form), and is sensitive to oxygen, pH, and temperature. Together with extensive first-pass metabolism, this poor aqueous solubility and photochemical instability give resveratrol low bioavailability and represent a formulation problem [7].

Resveratrol counteracts skin aging through several complementary routes, which act directly onto the photoaging cascade described above. First, its phenolic hydroxyl groups directly scavenge ROS; on top of this stoichiometric effect, resveratrol activates the Nuclear factor erythroid 2–related factor 2 (Nrf2)–Antioxidant response element (ARE) pathway and thereby up-regulates endogenous antioxidant enzymes such as superoxide dismutase, catalase, and glutathione related defenses. This provides a catalytic and self-renewing layer of protection against UV-induced oxidative stress [4,8,9]. Secondly, it is a well-characterized activator of SIRT1, a nikcotinamide adenine dinucleotide (NAD) dependent deacetylase linked to the beneficial effects of caloric restriction, mitochondrial function, and cellular longevity, which is thought to contribute to its protective effects in keratinocytes and fibroblasts [10,11]. Thirdly, it is a markedly anti-inflammatory molecule because it inhibits NF-κB signaling and lowers pro-inflammatory mediators (Tumor necrosis factor alpha (TNF-α), Interleukin 6 (IL-6), Cyclooxygenase-2 (COX-2)) [3,8,12]. Trans-resveratrol has been shown to inhibit hydrogen peroxide accumulation in human keratinocytes and to protect skin cells against UVA-induced damage. It also exhibits anti-melanogenic (tyrosinase-inhibiting) activity relevant to skin brightening [8,9,13]. Its antioxidant potency is frequently cited as being of the order of 50 times that of vitamin E and 30 times that of vitamin C on a comparative basis [14].

These mechanisms are explained in many areas of the dermatological and clinical literature. In cosmetic science, resveratrol is valued as a topical antioxidant and anti-aging active whose antioxidant, photoprotective, anti-inflammatory, and skin-brightening actions have been reviewed extensively [15]. Clinically, a topical antioxidant combining resveratrol with baicalin and vitamin E produced measurable improvements in fine lines, firmness, and photodamage [16], and a recent randomized, double-blind, placebo-controlled trial reported that oral plus topical trans-resveratrol significantly reduced visible wrinkle scores in women over 40 with good tolerability [17]. At the systemic level, the same molecule has shown cardiovascular, metabolic, neuroprotective, and anticancer activity in preclinical and early-phase clinical work [10,18,19,20]. In all of these important topical and systemic uses, the limitation is its delivery and bioavailability rather than any lack of activity [7,18].

To overcome these limitations, lipid-based vesicular carriers such as liposomes have been widely explored to stabilize and deliver dermacosmetic antioxidants, which help protecting unstable actives and providing controlled release [21,22]. Classical liposomes, however, can show limited entrapment of some actives and relatively rapid release, and they are not always very stable. Ethosomes are modified liposomal carriers first introduced by Touitou and co-workers [23]. Many researchers addressed limitations of liposomes and discussed ethosomes containing a relatively high ethanol content (approximately 20–50%), which acts as a skin penetration enhancer, reduces vesicle size, increases bilayer fluidity and deformability, and improves the entrapment of lipophilic and amphiphilic actives [23,24,25,26,27,28,29]. These properties make ethosomes particularly suitable for a poorly water-soluble, photolabile antioxidant such as trans-resveratrol, which can partition efficiently into, and be protected within, the ethanol–phospholipid bilayer.

The present work is a companion study to our previously published investigation of ethosomal nanocarriers for hydrolyzed collagen peptides [30], and follows the same formulation platform and characterization framework so that the actives can be compared directly. Although hydrolyzed collagen is a highly hydrophilic peptide, trans-resveratrol is a lipophilic small molecule. Therefore, the present study also examines how the same phospholipid–ethanol system accommodates a bilayer-partitioning active. Ethosomal delivery of trans-resveratrol has itself been reported before—for example, ethanol-containing ethosomes characterized for size, entrapment, HaCaT cytotoxicity and porcine skin permeation [31], a quality-by-design optimized resveratrol ethosomal hydrogel [32], and a resveratrol ethosomal cream for contact dermatitis [33]. The novelty of the present work therefore lies not in the concept but in the matched same-platform comparison of a lipophilic small molecule with our hydrophilic-peptide ethosome; the use of microfluidization with cycle number and pressure as the size-controlling variables for reproducible, scalable production; the 180-day physical- and encapsulation-stability dataset; and the release comparison across cellulose acetate and skin-mimicking Strat-M^®^ membranes. The aims of this study were to (a) develop and validate an HPLC assay method for trans-resveratrol, (b) prepare and optimize resveratrol-loaded ethosomes by varying pressure applied and the cycles of microfluidization process to easily produce and reduce the sizes of ethosomes, (c) characterize vesicle size, colloidal stability, encapsulation efficiency, morphology, cytotoxicity, and (d) investigate the *in vitro* release profile in order to evaluate ethosomes as a delivery platform for this antioxidant dermacosmetic active and also make an attempt to obtain preliminary information on the skin delivery behavior of resveratrol by repeating the Franz cell diffusion testing using the Strat M^®^ membrane.

## 2. Materials and Methods

The formulation platform, instrumentation, and characterization procedures used here are identical to those established for the earlier study of hydrolyzed collagen ethosome [30]. The resveratrol-specific materials, the HPLC assay (Shimadzu Corporation, Kyoto, Japan), and the points of difference are described in detail below.

### 2.1. Materials and Instrumentation

Trans-resveratrol was used as received; a certificate of analysis was provided by the supplier (CAS No: 501-36-0, Organic Herb Inc., Changsha, China). Ethanol (>99%, Merck, Darmstadt, Germany) and soybean lecithin containing phosphatidylcholine (Lipoid P75, Lipoid GmbH, Ludwigshafen am Rhein, Germany; >70% phosphatidylcholine) were used as the phospholipid source, and vitamin E (BASF, Ludwigshafen, Germany) was used as an antioxidant excipient. Disodium phosphate, monopotassium phosphate, sodium chloride, and potassium chloride (Merck/Sigma-Aldrich, Darmstadt, Germany) were used to prepare phosphate-buffered saline (PBS), and polysorbate 20 (Emulgin SML 20, BASF, Ludwigshafen, Germany) was added at 2% to the PBS receptor medium. Acetonitrile and formic acid (HPLC grade) were used for chromatography, and ultrapure water (Merck, Darmstadt, Germany) was used for all analyses. The XTT Cell Proliferation Kit (Roche Diagnostics, Mannheim, Germany) and DMEM (Sigma-Aldrich, Darmstadt, Germany) were used for cell viability assays and sodium lauryl sulphate (BASF, Ludwigshafen, Germany) used as control.

A high-pressure homogenizer (Microfluidizer M-110P, Microfluidics, Middleborough, MA, USA) was used for ethosome preparation and particle size reduction. Particle size and distribution were analyzed using a Mastersizer 2000 (Malvern Instruments, Malvern, UK), and zeta potential using an Anton Paar Litesizer 500 (Anton Paar, Graz, Austria). FT-IR spectra were collected on a Thermo Scientific Nicolet iS5 spectrometer with an iD5 ATR accessory (Thermo Fisher Scientific, Waltham, MA, USA). pH was measured with a WTW pH meter (Xylem/WTW, Weilheim, Germany) and density with an Anton Paar DMA 38 digital density meter (Anton Paar, Graz, Austria). Skin permeation experiments used a Logan FDC-6 Franz diffusion cell system (Logan Instruments, Somerset, NJ, USA); Strat-M^®^ synthetic membranes (Merck, Darmstadt, Germany) and cellulose acetate membranes with a 12–14 kDa molecular weight cut-off (Spectrum Laboratories, Rancho Dominguez, CA, USA) were used as diffusion membranes.

### 2.2. HPLC Assay and Validation of Trans-Resveratrol

Trans-resveratrol was quantified by HPLC (Shimadzu SIL-20AC HT autosampler and pump) on an Inertsil ODS-3 column (GL Sciences Inc., Tokyo, Japan, 250 mm × 4.6 mm, 5 µm). Mobile phase A was acetonitrile and mobile phase B was 0.2% formic acid, delivered as a gradient at 1.5 mL/min with a 40 °C column temperature; 20 µL of sample was injected and detection was at 306 nm. Test and reference solutions were prepared in ethanol (sonicated 10 min, cooled, made to volume) and filtered through 0.45 µm PTFE filters; calibration standards were prepared by dilution over the range 0.0075–0.25 mg/mL. The method was validated according to ICH Q2 (R1) [34] for linearity, accuracy, precision (repeatability), LOD, LOQ, range, robustness, and specificity, as detailed in the enclosed Supporting Information section. The method was validated using free trans-resveratrol standards in ethanol; specificity was confirmed against an unloaded ethosome dispersion, and the same validated method quantified free resveratrol in the ultracentrifugation supernatant for the encapsulation efficiency determination. The 0.45 µm PTFE filters were selected for their low non-specific binding, and the first portion of each filtrate was discarded.

Solubility of trans-resveratrol was determined by stirring 1 g of active in 30 mL of medium (ethanol, PBS pH 7.2, or PBS containing 2% polysorbate 20) at 150 rpm and 25 °C for 1 h, filtering through a 0.45 µm membrane, and assaying the filtrate by the HPLC method above; three replicates were performed for each medium.

### 2.3. Preparation of Resveratrol Ethosomes

Ethosomes were prepared by the cold method [30,35]. For the loaded formulations, 5% (*w*/*w*) Lipoid P75 and 0.3% (*w*/*w*) vitamin E were dissolved in 30% (*w*/*w*) ethanol under mechanical stirring (300 rpm), and 1.5% (*w*/*w*) trans-resveratrol was dissolved in this ethanolic lipid phase. The aqueous phase (deionized water, 63.2% *w*/*w*) was then added dropwise at 30 °C under continuous stirring to induce vesicle formation, and stirring was continued for a further 5 min; unloaded ethosomes were prepared identically, omitting the active and adjusting the water content to 64.7% (Table 1). Both loaded and unloaded dispersions were then passed through Microfluidizer M-110P for high-pressure homogenization and size reduction. The conditions used were 1000 bar pressure; 1, 3, 5, or 7 cycles (each cycle corresponding to a single pass through the interaction chamber), at 25 °C [36]. Within the interaction chamber, there are microchannels in which product stream is accelerate to high speeds and impinges itself on surfaces and particles splits into smaller particles.

### 2.4. Characterization of the Ethosomes

Particle size and distribution uniformity (laser diffraction; volume-weighted median diameter d_0.5_), zeta potential, pH, conductivity, density, and cryo-SEM morphology were determined exactly as described in the earlier study [30]. Physical stability was assessed by monitoring particle size, size distribution uniformity, zeta potential, and appearance at 1, 7, 14, 30, and 180 days at 25 ± 2 °C. FTIR spectra of loaded and unloaded ethosomes were compared to assess active–bilayer interactions. Stability was assessed on the neat ethosomal dispersions (the formulation medium containing 30% ethanol; no external buffer was added), stored at 25 ± 2 °C.

### 2.5. Encapsulation Efficiency

Encapsulation efficiency was determined by ultracentrifugation of the ethosome dispersion followed by HPLC quantification of free (unencapsulated) resveratrol in the supernatant. EE (%) was calculated as shown in Equation (1). Because EE was obtained indirectly from the free resveratrol measured in the supernatant, it does not capture active lost to vessel surfaces or to degradation during processing; a direct assay of resveratrol recovered from the sedimented lipid phase would refine the value. However, this method is a commonly used method for determining EE.

EE = (T − C)/T × 100(1)

where T is the total amount of resveratrol added to the formulation and C is the unentrapped amount detected in the supernatant [30].

### 2.6. In Vitro and Biological Evaluation

Cytotoxicity was evaluated in HaCaT keratinocytes using the XTT Cell Proliferation Assay following ISO 10993-5 [37], with formulations and pure resveratrol tested at 25, 10, 1, and 0.1%, sodium lauryl sulfate as positive control and culture medium as negative control.

In vitro release was assessed using a Franz diffusion cell system with cellulose acetate (12–14 kDa) and Strat-M^®^ membranes (0.21 cm^2^); 1 mL of ethosomal dispersion or 1.5% resveratrol solution was used as the donor compartment, and receptor aliquots were withdrawn at 1, 2, 4, 8, 16, 24, and 48 h and quantities were determined by HPLC. A 2% polysorbate-20 PBS solution was used as the receptor medium, at each sampling time 1 mL of warmed receptor solution was added to replace the sample withdrawn to maintain sink condition also to ensure the receptor solution making full-surface contact with the membrane without any air bubbles [30].

### 2.7. Statistical Analysis

All experiments were performed in at least three replicates, and results are expressed as mean ± standard deviation (SD). Where applicable, group differences were assessed by one-way analysis of variance (ANOVA) followed by Tukey’s test, with *p* < 0.05 considered statistically significant.

## 3. Results and Discussion

### 3.1. Analytical Method Validation of the Trans-Resveratrol Assay

#### 3.1.1. Identity Confirmation by FT-IR

**Figure 1:**
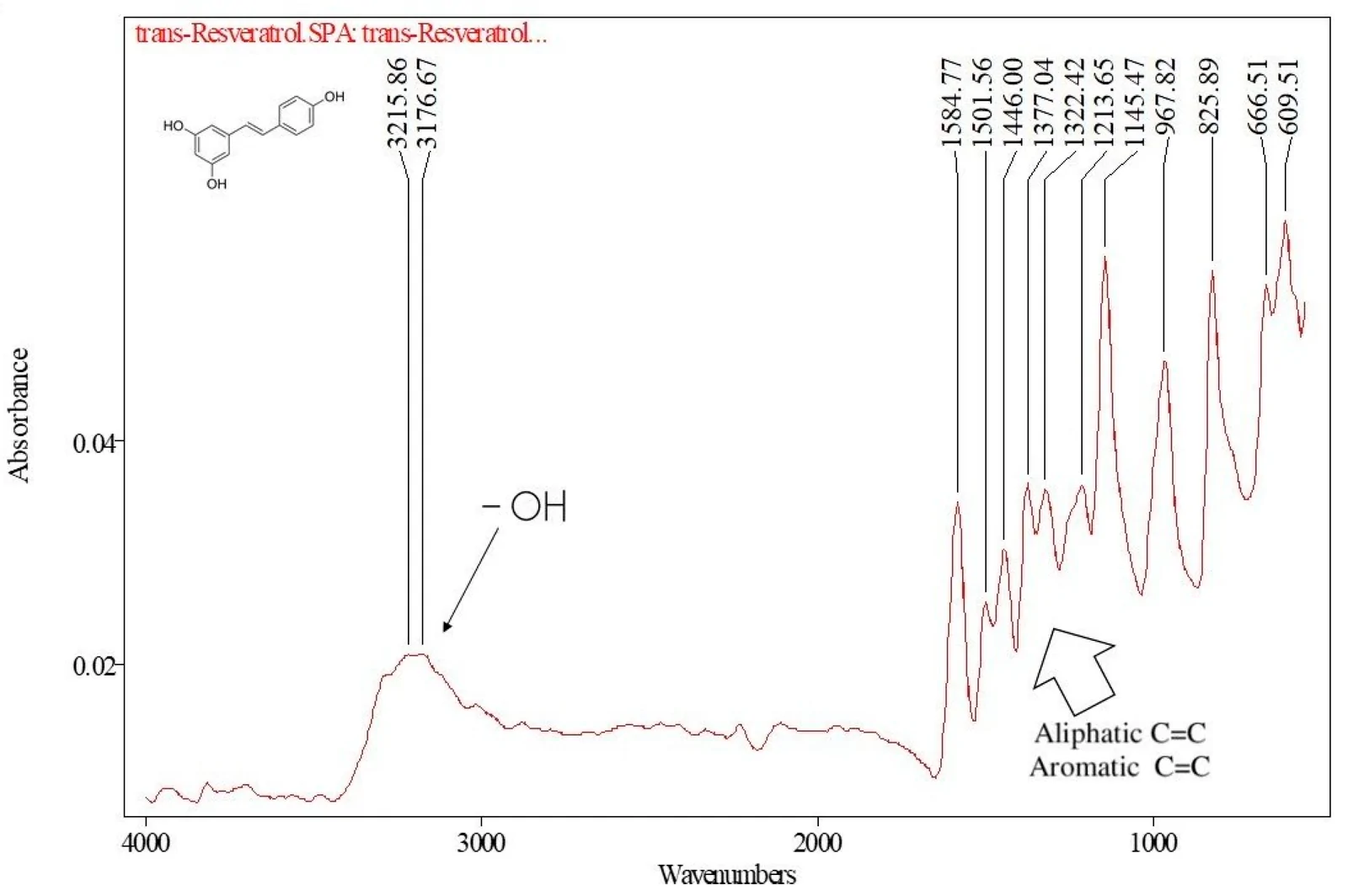
FT-IR, Fourier-transform infrared spectroscopy. FT-IR spectrum of trans-resveratrol showing the characteristic O–H and aromatic/aliphatic C=C absorption bands.

The identity of trans-resveratrol was confirmed by Fourier-transform infrared (FT-IR) spectroscopy (Figure 1). The spectrum showed the characteristic broad O–H stretching bands at 3215 and 3177 cm^−1^ arising from the three phenolic hydroxyl groups, together with aromatic and aliphatic C=C stretching and stilbene-backbone bands at 1584, 1502, 1446, 1377, 1322, 1214, 1145, 968, 826, 666, and 609 cm^−1^. These bands are consistent with reference spectra of trans-resveratrol and confirm the chemical integrity of the active before formulation.

#### 3.1.2. HPLC Method Validation

**Figure 2:**
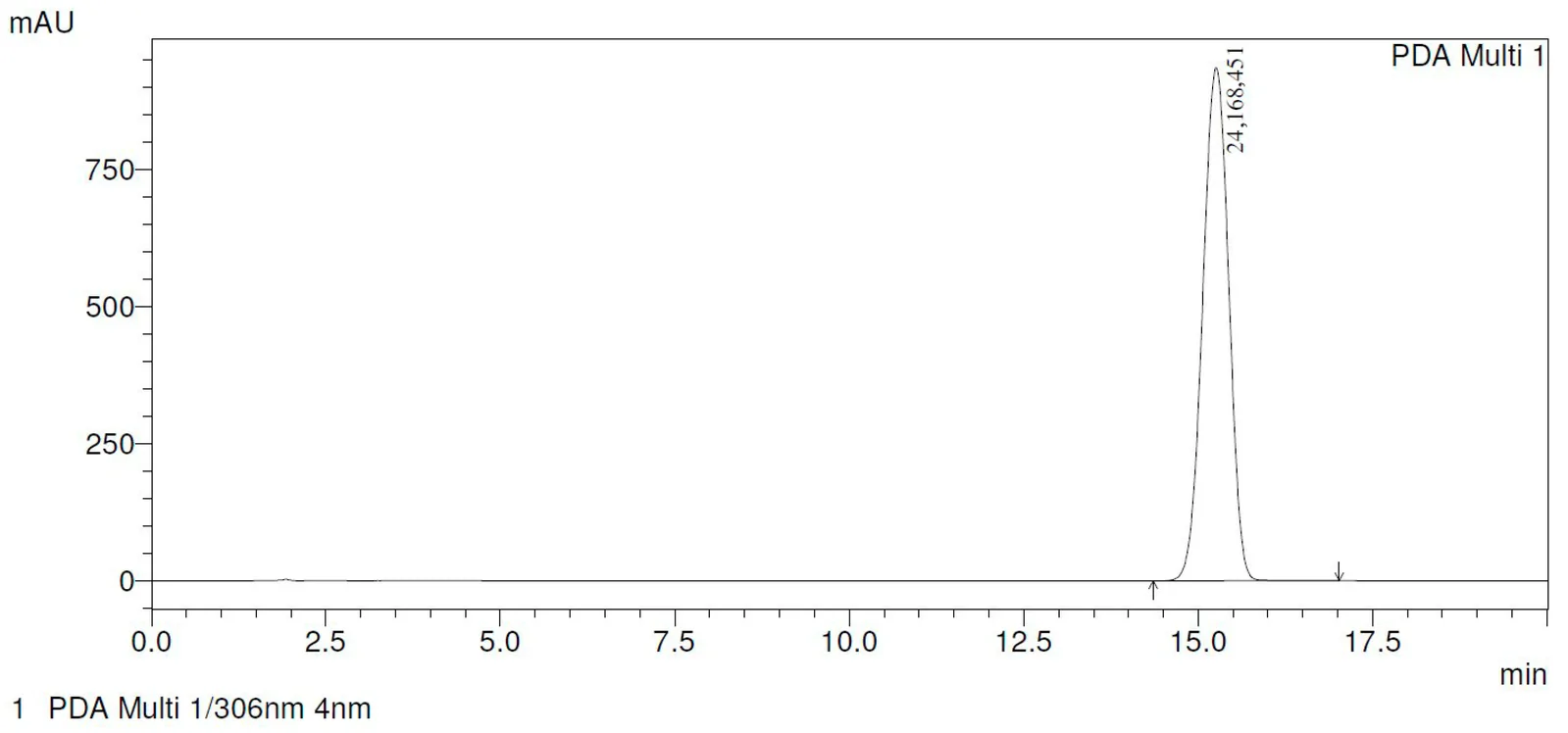
HPLC, high-performance liquid chromatography. Representative HPLC chromatogram of trans-resveratrol (306 nm); a single, well-resolved peak confirms method specificity.

A reversed phase HPLC method was developed for trans-resveratrol (Inertsil ODS-3 column; acetonitrile/0.2% formic acid gradient; 1.5 mL/min; 40 °C; detection at 306 nm) and validated according to ICH Q2 [34] for linearity, accuracy, precision, limit of detection (LOD), limit of quantitation (LOQ), robustness, and specificity. Linearity over six concentration levels gave a regression equation y = 1 × 10^8^x + 430,677 and R^2^ = 0.9994, exceeding the R^2^ ≥ 0.998 criterion (Figure S1). Accuracy at 21, 50, and 100 µg/mL gave mean recoveries of 102.8–104.2%, within the accepted 90–110% range. Repeatability at 10, 20, and 110 µg/mL and at 220 µg/mL gave coefficients of variation below 2%. The LOD and LOQ were 4.8 and 14.5 µg/mL, respectively. Robustness testing of a 200 µg/mL sample over 24 h showed only 0.83% variation, and specificity was confirmed by the absence of interfering peaks from unloaded ethosomal formulations at the resveratrol retention time (around 15.2 min; Figure 2). Full validation data is provided in the Supplementary Materials.

Solubility studies defined the medium in which resveratrol can actually be dissolved and quantified, and therefore shaped the practical formulation decisions. Resveratrol was highly soluble in ethanol (65 mg/mL) but only sparingly soluble in phosphate-buffered saline (PBS, pH 7.2; 100 µg/L), increasing to about 500 µg/L in PBS containing 2% polysorbate 20. Because resveratrol dissolves readily in ethanol, the ethanol phase of the ethosome not only carries the active into the phospholipid bilayer and the vesicle core but also acts as a penetration enhancer.

Because resveratrol is almost insoluble in aqueous buffer, a simple PBS receptor medium would not maintain the sink conditions in the release experiments. The solubility data therefore indicated that a surfactant-containing receptor medium was required for the Franz cell studies; the 2% polysorbate-20 PBS medium was selected to keep released resveratrol in solution and allow a valid release measurement.

### 3.2. Formulation, Particle Size, and Stability of Resveratrol Ethosomes

Resveratrol-loaded ethosomes were prepared following the cold method established earlier [30], with the lipophilic active dissolved in the ethanolic phospholipid phase prior to hydration, and then processed by high-pressure homogenization interaction chamber (M110P) with one, three, five, or seven cycles. All resveratrol-loaded formulations contained 5% lecithin (Lipoid P75), 30% ethanol, 0.3% vitamin E, and 1.5% trans-resveratrol; by using the same method and identical conditions, loaded/unloaded ethosomes were prepared. Compositions are summarized in Table 1. The 1.5% (*w*/*w*) resveratrol load was set from the solubility of resveratrol in the ethanolic lipid phase (65 mg/mL in ethanol), keeping the active fully dissolved and below saturation before hydration, while remaining within the range typical for cosmetic use.

**Figure 3:**
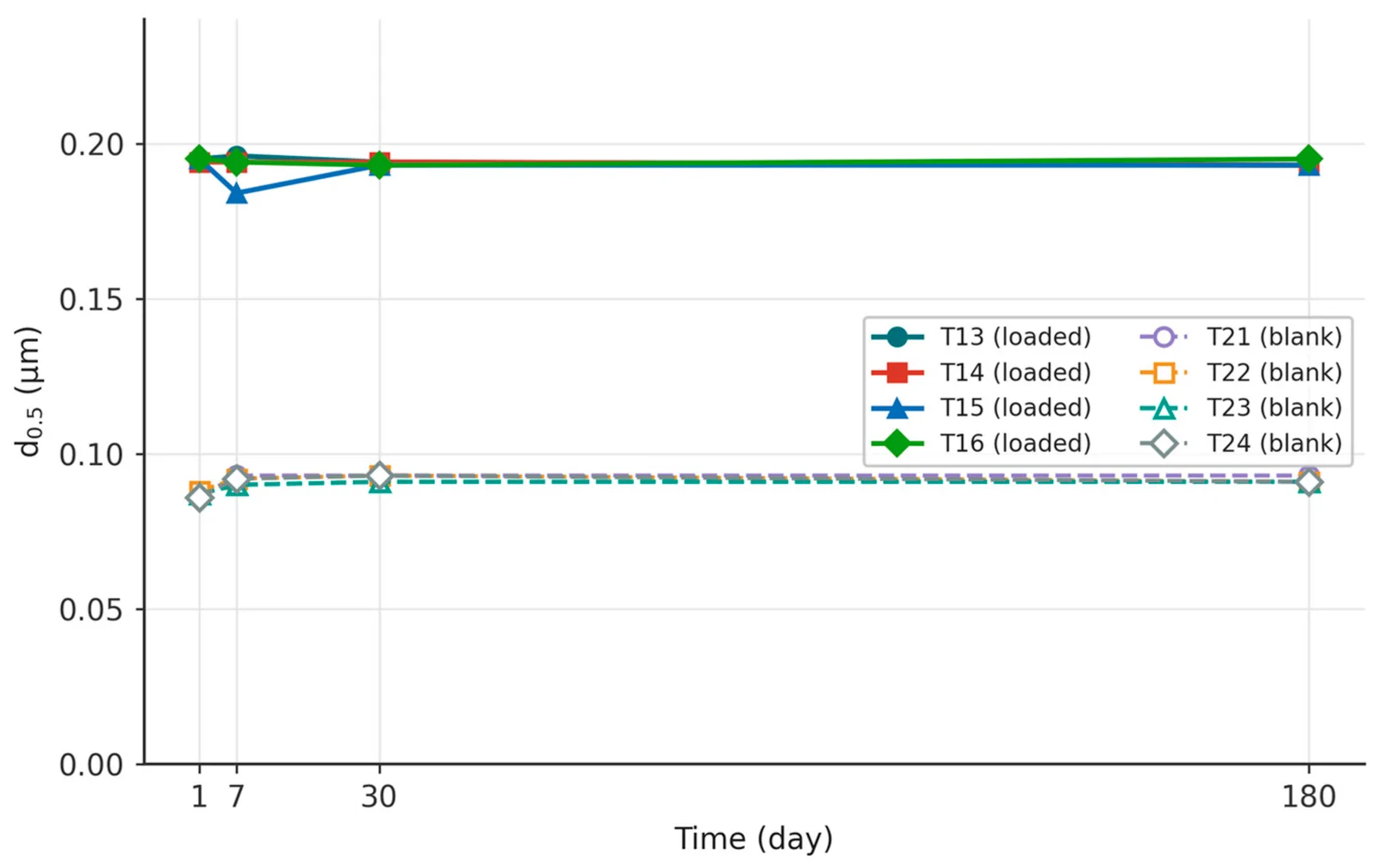
Particle size (d_0.5_) of resveratrol-loaded (T13–T16, solid lines/filled symbols) and unloaded (T21–T24, dashed lines/open symbols) ethosomes over 180 days at 25 ± 2 °C; d_0.5_, volume-weighted median diameter.

**Figure 4:**
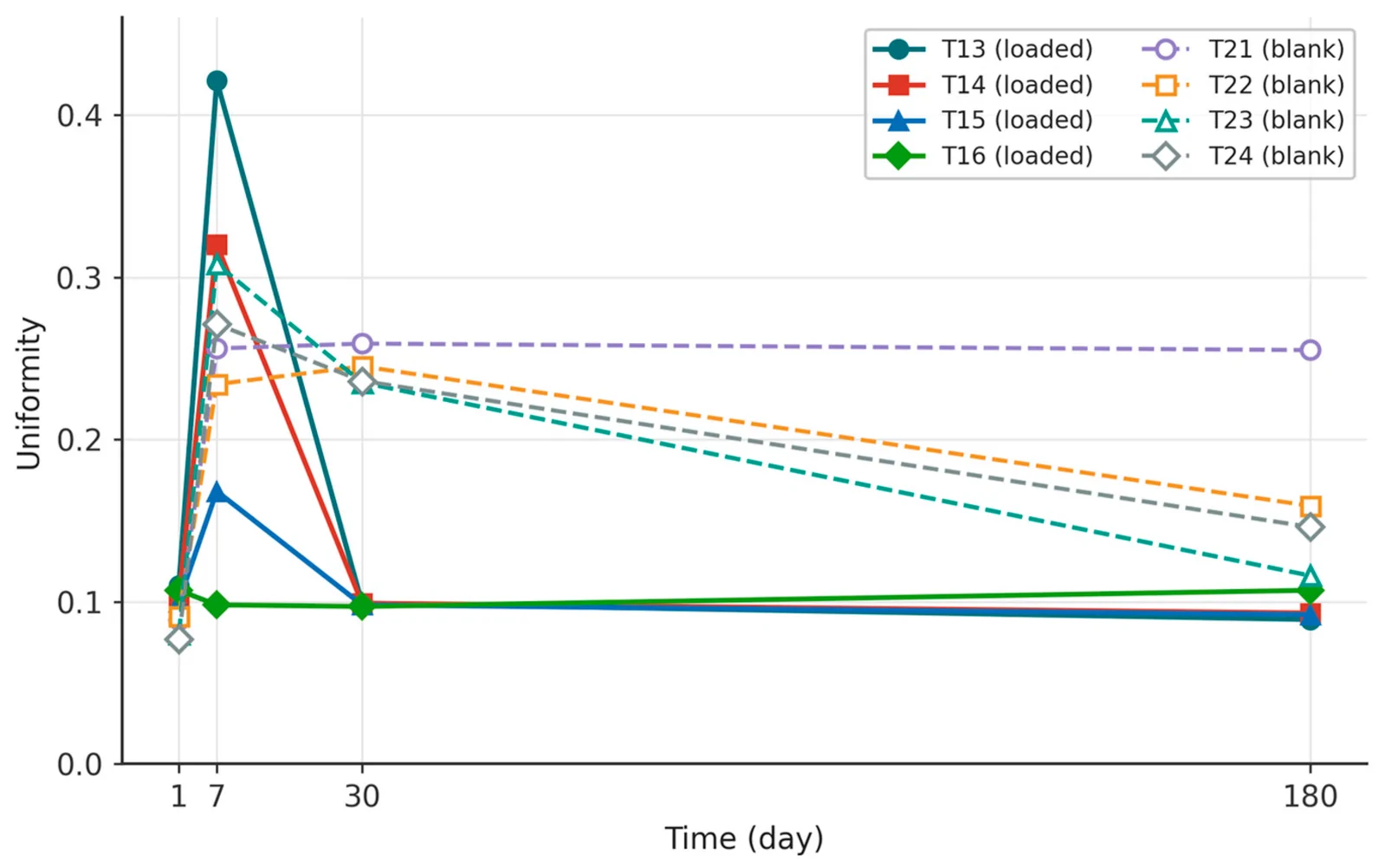
Size distribution uniformity of resveratrol-loaded (T13–T16, solid lines/filled symbols) and unloaded (T21–T24, dashed lines/open symbols) ethosomes over 180 days.

Particle size and distribution uniformity were monitored by laser diffraction over 180 days (Table 2). The resveratrol-loaded ethosomes (T13–T16) formed a homogeneous nanosized population with volume-weighted median diameters (d_0.5_) of approximately 0.19 µm (190 nm) that remained essentially unchanged over the study period, indicating good physical stability with no significant aggregation or phase separation. The larger diameter of the loaded vesicles relative to the unloaded ethosomes (T21–T24) (average 90 nm) is consistent with accommodation of the lipophilic active within the phospholipid bilayer. Increasing the number of homogenization cycles helped to improve the size distribution uniformity. Formulation T16 (seven cycles) had a consistent size and low, stable uniformity at all time points tested. Formulation T16, along with the unloaded T24, was used in the later encapsulation, morphology, and release studies. Size and uniformity are shown in separate figures (Figure 3 and Figure 4). In this study, “distribution uniformity” is the dimensionless distribution-width parameter reported by the Malvern Mastersizer laser-diffraction software (uniformity = Σ X_i_|d(v,0.5) − d_i_|/[d(v,0.5) Σ X_i_]); it is distinct from the dynamic-light-scattering polydispersity index, and lower values indicate a narrower distribution. Because mean diameter and uniformity alone cannot exclude aggregation, physical stability was judged from the combination of a constant d_0.5_, stable uniformity, retained zeta potential, and visual inspection for creaming, sedimentation, or surface-film formation at each time point product was tested.

### 3.3. Physicochemical Properties and Colloidal Stability

**Figure 5:**
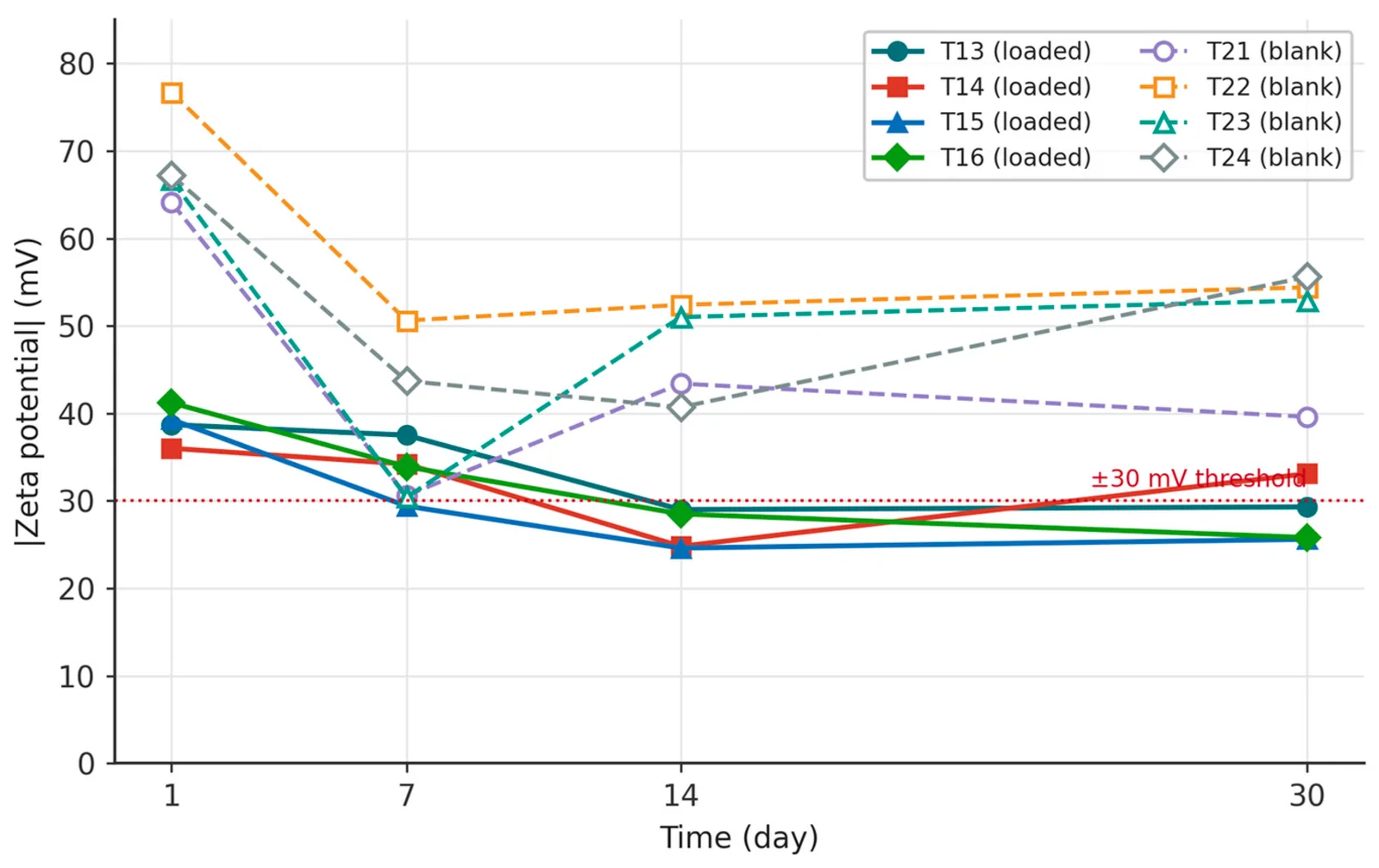
Absolute zeta potential of resveratrol-loaded (T13–T16, solid) and unloaded (T21–T24, dashed) ethosomes over 30 days; the dotted line marks the 30 mV colloidal stability threshold.

The pH, conductivity, density, and zeta potential of the resveratrol-loaded (T13–T16) and unloaded (T21–T24) ethosomes are summarized in Table 3 and Figure 5. The pH of the loaded formulations (6.50–6.54) is close to the physiological pH of skin and therefore suitable for topical application, while conductivity (44.4–44.6 µS/cm) and density (0.949–0.961 g/cm^3^) were consistent across formulations, indicating uniform, reproducible dispersions. Zeta potential is a one of the key indicators of colloidal stability, with absolute values above 30 mV generally indicating good electrostatic stabilization [38]. Initial absolute zeta potentials of the loaded formulations were 38.7, 36.0, 39.3, and 41.2 mV for T13–T16, and although a gradual decrease was observed over storage, no visible aggregation or phase separation occurred. Conductivity was recorded as a simple, reproducible indicator of the aqueous continuous phase and of batch-to-batch consistency. The consistent conductivity values, together with the stable pH and density shows the water-continuous system is maintained, and there is no indication of phase inversion or excessive coalescence.

Notably, the unloaded ethosomes exhibited consistently higher absolute zeta potentials than the loaded systems. The zeta potential of phospholipid ethosomes is negative, arising from the anionic phosphate groups of the phosphatidylcholine head-groups; incorporation of resveratrol lowers the magnitude of this surface charge.

Most likely some polyphenol molecules move into the outer bilayer, where its hydroxyl groups form hydrogen bonds with and partly shield the phosphate charges on the vesicle surface, which lowers the measured zeta-potential. Similar decreases in zeta potential after drug loading have been reported: Verma and Pathak observed −48.8 to −75.1 mV for econazole-loaded ethanolic vesicles [39], and Al-Ameri and Al-Gawhari reported −36.3 to −51.0 mV for soya lecithin ethosomes [40], matching the range and the loading-related decrease in our experiments [38,39,40].

### 3.4. Encapsulation Efficiency

The encapsulation efficiency (EE) of the optimized resveratrol ethosome (T16) was determined by HPLC quantification of free actives in the supernatant after ultracentrifugation, following the method described in our earlier study [30] (Table 4). On day 1, T16 exhibited a very high EE of 95.5% (4.5% unencapsulated), markedly higher than the 86% obtained for the hydrophilic collagen peptide ethosomes prepared by the same method [30]. This high entrapment is consistent with the lipophilic character of trans-resveratrol, which partitions efficiently into the ethanol-fluidized phospholipid bilayer rather than residing in the aqueous core. After 180 days of storage, EE decreased moderately to 84.2%, retaining the majority of the encapsulated active; such gradual reductions are commonly reported for phospholipid vesicular systems and attributed to slow bilayer rearrangement and diffusion during prolonged storage. Retention of >84% of the active after six months indicates that the ethosomal bilayer provides an effective and stable reservoir for resveratrol.

### 3.5. Morphological and Molecular Characterization

#### 3.5.1. Cryo-SEM Analysis

**Figure 6:**
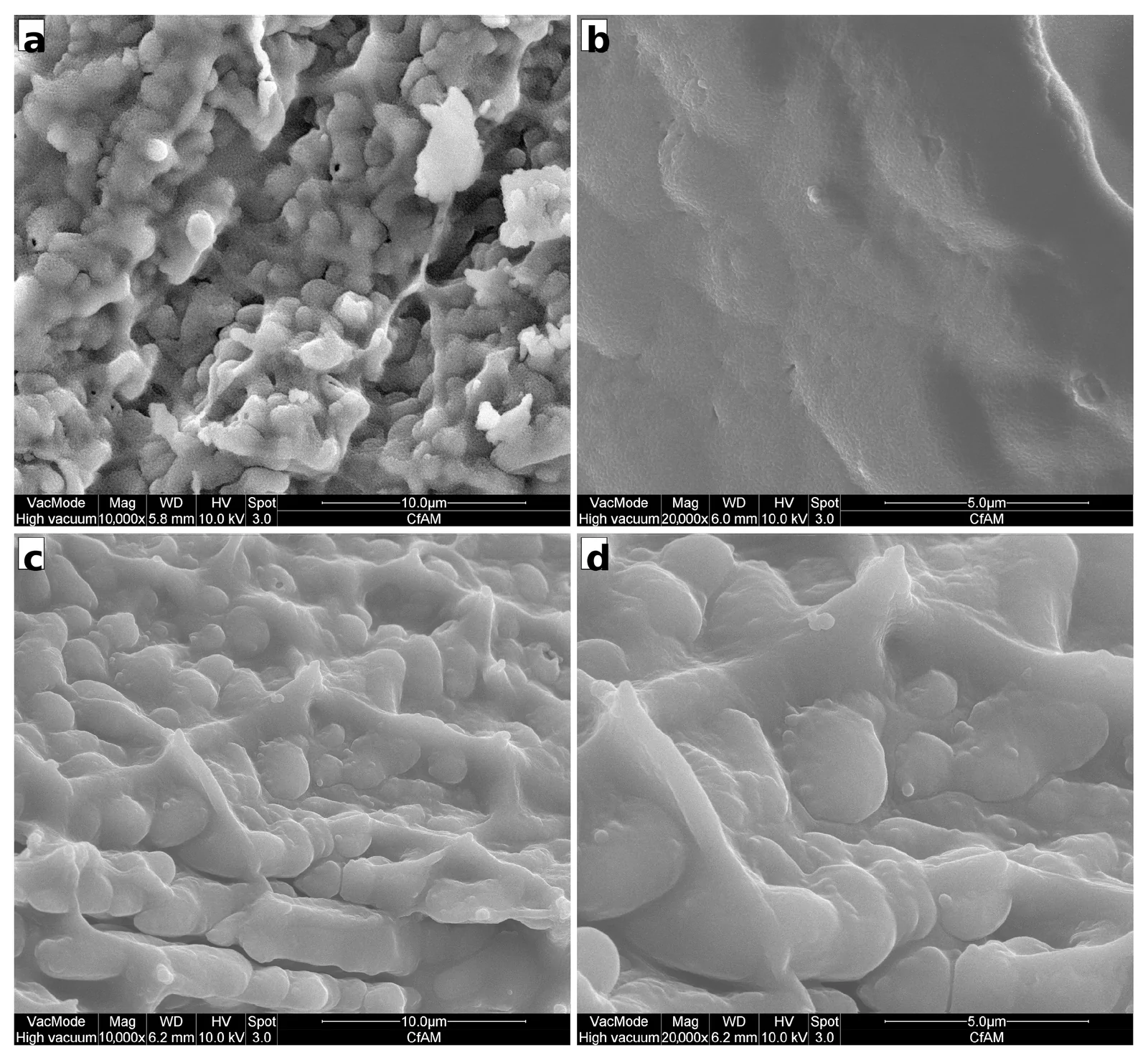
Cryo-SEM images of (**a**,**b**) unloaded ethosomes (T24; sample BF0C1B1000) at 10 µm and 5 µm and (**c**,**d**) resveratrol-loaded ethosomes (T16; sample BF1C5B1000) at 10 µm and 5 µm. Original, uncropped micrographs are presented (Centre for Advanced Microscopy, University of Reading) in the Supplementary Materials.

Vesicle morphology was examined by cryo-scanning electron microscopy (cryo-SEM), which preserves the native hydrated state of the vesicles and minimizes the structural collapse often seen with conventional SEM. The unloaded (T24) and loaded (T16) ethosomes both exhibited generally spherical to near-spherical morphology, confirming successful vesicle formation (Figure 6a–d). Quantitative morphometry (Table 5) found a mean aspect ratio of 1.43 for resveratrol-loaded vesicles and 1.37 for the unloaded, which is a small deviation from perfect spheres that may come from interactions between the bilayer and resveratrol. Both formulations had very low image-based size dispersion (PDI 0.038 and 0.026), showing narrow and uniform shapes. This is common with cryo-fixation of phospholipid vesicles; SEM diameters appear larger than median diameters from laser diffraction because of ice-crystal growth and 2D projection during sample preparation that inflate sizes; a similar SEM vs. DLS difference has been reported previously [41]. The unloaded (T24) and resveratrol-loaded (T16) ethosomes were imaged as cryo-SEM sample codes BF0C1B1000 and BF1C5B1000, respectively; the original micrographs are provided in the Supplementary Materials.

Vesicle morphology was quantified uniformly across the panels of Figure 6. Each measured vesicle is described by the same descriptors: the projected area (µm^2^, from image analysis) and the derived equivalent circular diameter (d = 2√(area/π)), together with the aspect ratio. As a result, identical metrics are now reported for the unloaded (panels a, b; for T24) and resveratrol-loaded (panels c, d; for T16) ethosomes. The results are summarized in Table 6. The vesicles were measured on the annotated cryo-SEM micrographs contained in the cryo-SEM report (Supplementary Materials), in which each vesicle is individually outlined and its projected area is labelled directly on that vesicle; therefore, every area value is unambiguously assigned to a specific vesicle.

Both vesicle populations were near-spherical and similarly shaped, with comparable aspect ratios (1.37 ± 0.22 for T24 versus 1.43 ± 0.28 for T16) and low image-based dispersion (0.026 versus 0.038). The resveratrol-loaded vesicles were larger than the unloaded ones (mean projected area 3.15 ± 1.26 versus 1.74 ± 0.56 µm^2^; equivalent circular diameter 1.96 ± 0.42 versus 1.47 ± 0.24 µm), consistent with accommodation of the lipophilic active resveratrol within the phospholipid bilayer. These cryo-SEM projected sizes are slightly larger than the laser-diffraction median (average 190 nm) because of ice-crystal growth and the two-dimensional projection of clustered vesicles during cryo-fixation. Cryo-SEM is therefore used here to confirm vesicle morphology and relative differences rather than to provide absolute hydrodynamic sizes.

#### 3.5.2. FTIR Spectroscopy of the Formulations

**Figure 7:**
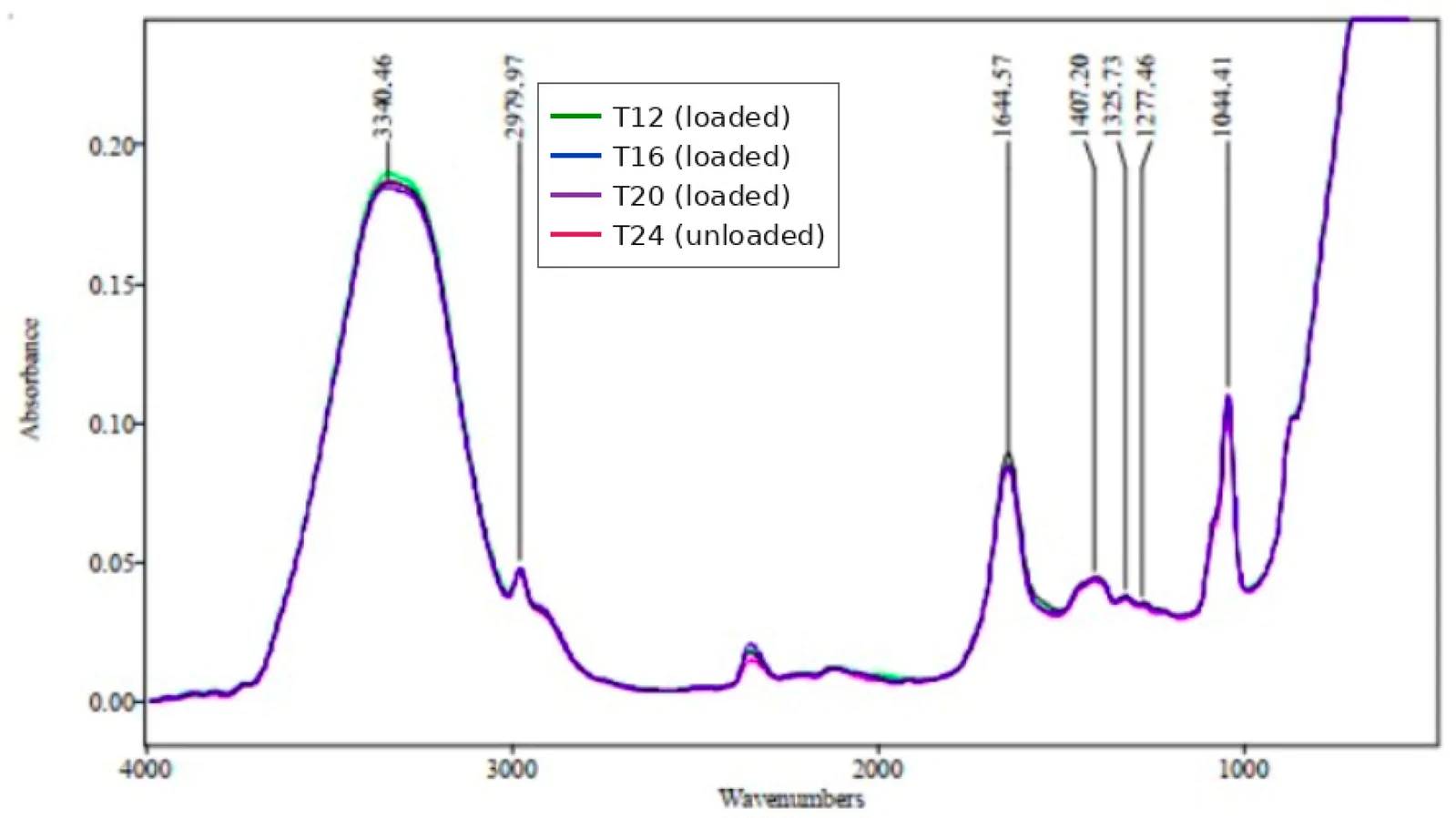
Comparative FT-IR spectra of the unloaded ethosome (T24) and the resveratrol-loaded ethosomes (T12, T16, T20). The spectra are essentially superimposable, showing phospholipid-dominated band patterns and no new covalent bands upon resveratrol loading. FT-IR, Fourier-transform infrared spectroscopy.

FTIR was used to probe whether resveratrol is chemically altered on encapsulation and whether it perturbs the phospholipid bilayer, by comparing the loaded ethosome with the corresponding empty (unloaded) ethosome (Figure 7). The spectra of the loaded and empty vesicles were essentially superimposable and were dominated by phospholipid-associated bands—a broad O–H/N–H envelope at ~3340 cm^−1^, CH_2_/CH_3_ stretching at 2980 cm^−1^, a carbonyl/amide-region band at ~1645 cm^−1^, and phosphate- and ester-related vibrations at 1407, 1326, 1277, and 1044 cm^−1^. Critically, no characteristic lipid peaks were lost, and no new covalent bands appeared on loading resveratrol, and the loaded spectrum did not differ materially from the unloaded ethosome spectrum, indicating that resveratrol did not chemically modify the bilayer. The absence of distinct resveratrol bands in the loaded spectrum is expected because the strong lipid-matrix signals dominate and mask the weaker absorbance of the entrapped active. FTIR therefore confirms preservation of the bilayer and non-covalent (physicochemical) association between resveratrol and phosphatidylcholine. Encapsulation itself is also demonstrated by the encapsulation efficiency and release data rather in addition to FTIR. This behavior, and the interpretation that entrapment occurs through non-covalent, physicochemical partitioning, are consistent with FTIR reports for other bioactive-loaded liposomal and ethosomal systems [42,43,44] and with the companion collagen study [30].

### 3.6. In Vitro and Biological Evaluation

#### 3.6.1. Cytotoxicity Assay (HaCaT Keratinocytes)

**Figure 8:**
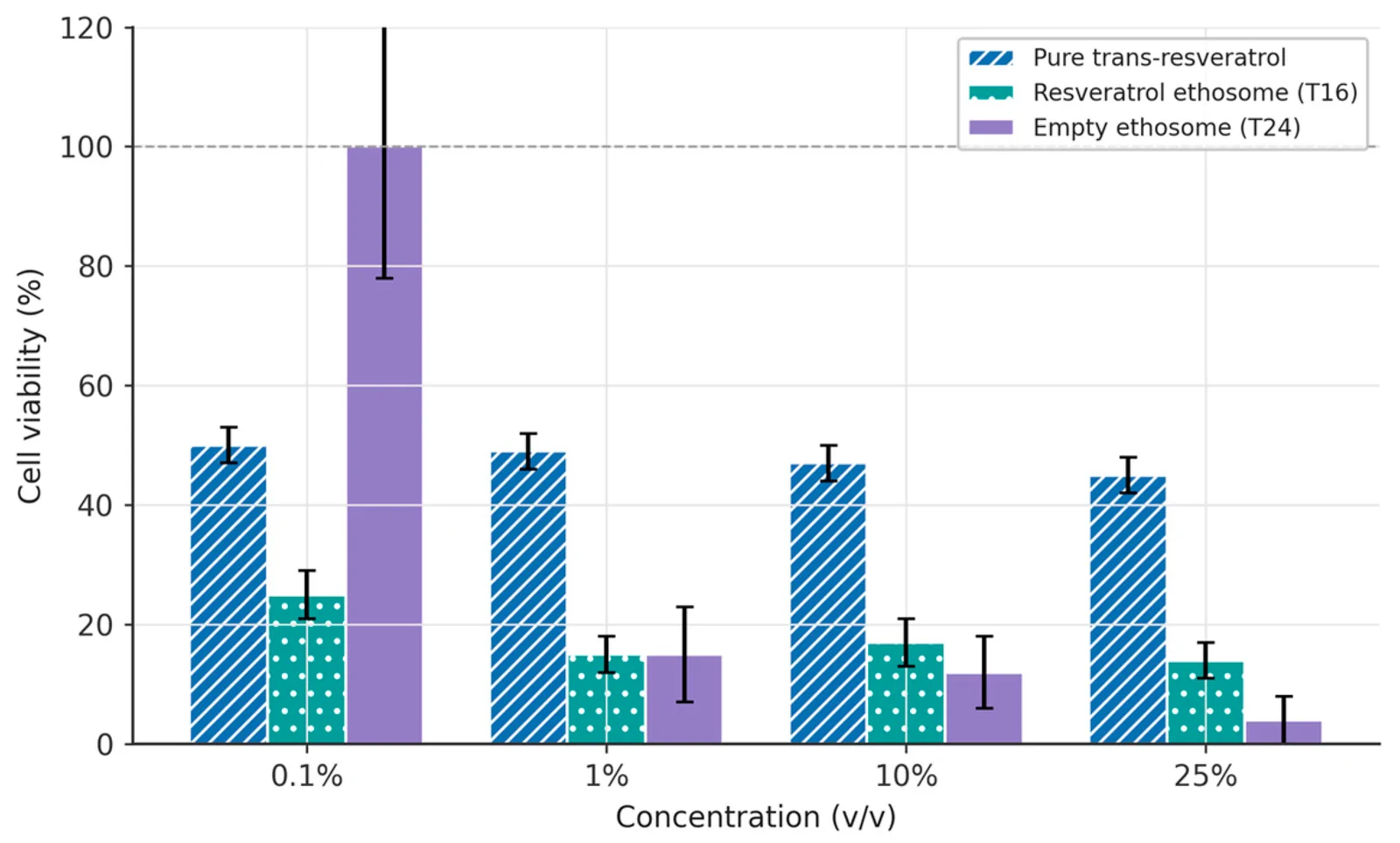
HaCaT (human keratinocyte cell line) keratinocyte viability after 24 h exposure to pure trans-resveratrol, the resveratrol-loaded ethosome (T16), and the empty ethosome (T24) at 0.1–25% (*v*/*v*); mean ± SD. SD, standard deviation; *v*/*v*, volume per volume.

The cytotoxicity of pure trans-resveratrol, resveratrol-loaded ethosomes (T16), and unloaded ethosomes (T24) was evaluated in HaCaT keratinocytes using the XTT viability assay over a wide concentration range (0.1–25%, *w*/*w*; Figure 8). These are high test concentrations relative to the levels at which resveratrol is used in finished topical products. Pure trans-resveratrol reduced keratinocyte viability to approximately 45–50% across the tested range, with only a weak concentration dependence, indicating a moderate intrinsic cytotoxicity of resveratrol at these concentrations. The empty ethosome (T24) was strongly concentration dependent: viability was essentially complete (around 100%) at 0.1% but fell sharply at higher concentrations (to 12% at 10% and 4% at 25%). The resveratrol-loaded ethosome (T16) showed reduced viability across the whole range (14–25%), combining the intrinsic effect of the active with the carrier effect, and recovered only partially (to around 25%) at the lowest concentration. These 0.1–25% (*w*/*w*) levels are one-to-three orders of magnitude above the realistic in-use concentrations; because the blank ethosome (T24) was tested in parallel, the effect is due mainly to the carrier (ethanol and the surfactant-like action of the vesicles at high dose), with resveratrol adding some more effect. Tolerability testing at cosmetic-use levels, ideally in a reconstructed human epidermis model, is the appropriate next step. The positive (sodium lauryl sulfate) and negative (culture medium) controls behaved as expected (Figure 8).

The cytotoxicity observed at high test concentrations is driven mainly by the ethosomes, while the loaded system additionally reflects the moderate intrinsic cytotoxicity of resveratrol. As discussed for the companion collagen ethosomes [30], the reduced viability of the vesicles at elevated concentrations is most possibly related to the ethanol content and the surfactant-like membrane action of the phospholipid vesicles, which can disrupt cell membranes at high doses; consistent with this, the empty carrier was well tolerated only at the lowest concentration. A similar dose-dependent pattern—minimal cytotoxicity at low doses and reduced viability at higher doses or longer exposure—has been reported for other ethosomal systems, for example, Gossypin-loaded ethosomes on melanoma cells [45]. These findings underscore that appropriately low-use concentrations, and further optimization to improve keratinocyte tolerance, are important for topical application. However, the results should be interpreted cautiously because the test concentrations were higher than what would occur naturally in the body. The control experiments performed as expected, which supports the reliability of the measurements.

#### 3.6.2. In Vitro Release Using Franz Diffusion Cells

**Figure 9:**
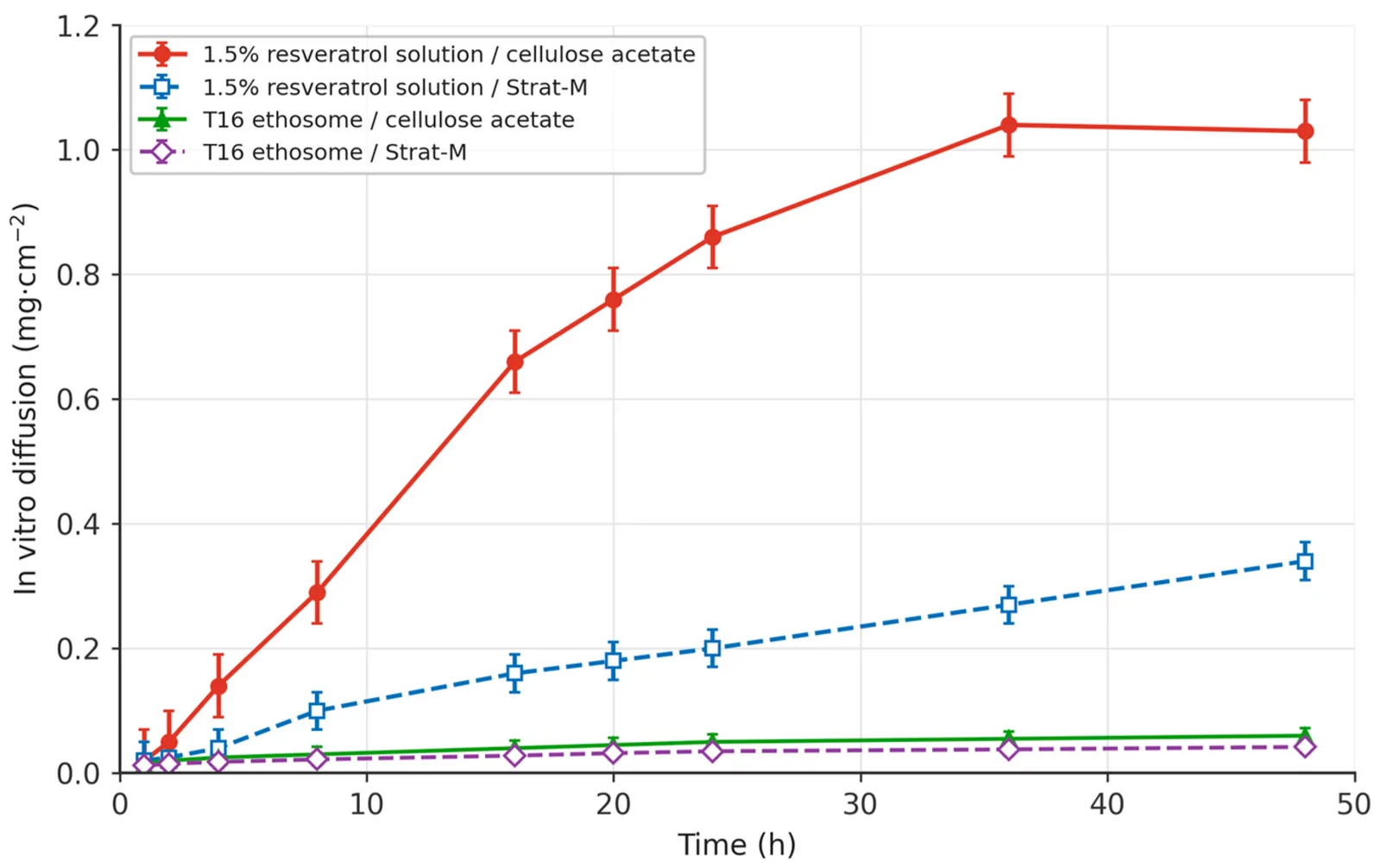
Time-dependent in vitro release of trans-resveratrol from the T16 ethosome versus a 1.5% *w*/*w* resveratrol solution across cellulose acetate and Strat-M membranes (Franz diffusion cells; mean ± SD). SD, standard deviation.

The in vitro release of trans-resveratrol from the optimized ethosome (T16) was evaluated using Franz diffusion cells fitted with either a cellulose acetate dialysis membrane (12–14 kDa) or a skin-mimicking Strat-M membrane, effective area of the cells was 0.21 cm^2^); a 2% polysorbate-20 PBS receptor solution was selected as receiving medium on the basis of the solubility data. A 1.5% resveratrol solution served as the free active comparator; the cumulative amount diffused per unit area was measured over 1–48 h (Figure 9). A consistent rank order was obtained: the free 1.5% solution diffused fastest across cellulose acetate, reaching around 1.03 mg/cm^2^ at 48 h, followed by the solution across Strat-M (0.34 mg/cm^2^), then the loaded ethosome across cellulose acetate (0.06 mg·cm^−2^), with the loaded ethosome across Strat-M the slowest 0.04 mg/cm^2^). The encapsulated resveratrol was released in a sustained manner compared to trans-resveratrol solution. There was almost an order-of-magnitude reduction in the amount diffused which is consistent with strong partitioning of the active into the ethanol–phospholipid bilayer and in the core of ethosome. Release was followed up to 48 h; the ethosome release profiles did not plateau but continued to rise slowly, reflecting bilayer retention rather than limited release. The slower diffusion across Strat-M relative to cellulose acetate reflects the higher barrier effect of the skin-mimicking membrane against resveratrol, a barrier that governs vesicle-mediated transdermal permeation [46,47,48,49]. From a dermacosmetic perspective, this prolonged availability of resveratrol at the skin surface or within the upper skin layers is advantageous and is consistent with the sustained-release behavior reported for ethosomal systems [26,30].

## 4. Conclusions

In this study, ethosomal formulations were successfully developed for the delivery of trans-resveratrol, a poorly water-soluble, antioxidant dermacosmetic active. Using the same phospholipid–ethanol platform established for hydrolyzed collagen peptides [30], stable nanoscale vesicles (190 nm) were obtained by microfluidization under high-pressure homogenization. A validated HPLC assay enabled reliable quantification of resveratrol. The optimized formulation (T16) achieved a very high encapsulation efficiency (95.5% on day 1, 84.2% after 180 days), consistent with efficient partitioning of the lipophilic active into the ethanol-fluidized bilayer and notably higher than for the hydrophilic collagen load. Comprehensive characterization—particle size, zeta potential, pH, conductivity, density, and cryo-SEM—demonstrated uniform, physically stable vesicles over 180 days, and FTIR confirmed preservation of the phospholipid bilayer, with encapsulation of resveratrol as non-covalent bilayer partitioning. In vitro Franz cell studies showed that ethosomal encapsulation provides a markedly sustained, controlled release compared to a simple resveratrol solution. Cell viability testing showed dose-dependent cytotoxicity driven mainly by the vesicular carrier at high test concentrations, underscoring the importance of appropriate use levels. Overall, it was shown that optimized trans-resveratrol-loaded ethosomes are an effective, stable nanocarrier for this antioxidant active, complementing the hydrophilic peptide system reported previously and supporting ethosomes as a versatile platform for anti-aging dermacosmetic ingredients. The microfluidization method proved to be a simple, reproducible method to stable average 190 nm ethosomes, with narrow size distribution, stable in the storage (180 days tested). Several limitations of this study should be noted: the photostability protection conferred by encapsulation was not directly measured and is the key confirmatory experiment for future work; the antioxidant and SIRT1/Nrf2/NF-κB/MMP-related activities highlighted in the Introduction were not tested in the loaded formulation; and release was assessed only with artificial membranes (cellulose acetate and Strat-M), so ex vivo and in vivo skin studies are required to confirm dermal delivery.

## Figures and Tables

**Table 1:** Composition of resveratrol-loaded (T13–T16) and unloaded (T21–T24) ethosome formulations. The optimized formulation (T16, bold) was carried forward for detailed characterization.

Type	Code	Water (%)	Lecithin (%) (Lipoid P75)	Vitamin E (%)	Ethanol (%)	Resveratrol (%)	Cycle *
Loaded	T13	63.2	5	0.3	30	1.5	1
Loaded	T14	63.2	5	0.3	30	1.5	3
Loaded	T15	63.2	5	0.3	30	1.5	5
Loaded	T16	63.2	5	0.3	30	1.5	7
Unloaded	T21	64.7	5	0.3	30	–	1
Unloaded	T22	64.7	5	0.3	30	–	3
Unloaded	T23	64.7	5	0.3	30	–	5
Unloaded	T24	64.7	5	0.3	30	–	7

* Number of passes through the high-pressure homogenizer interaction chamber (Microfluidics M110P).

**Table 2:** Vesicle size (d_0.5_) and distribution uniformity of resveratrol-loaded and unloaded ethosomes over 180 days (25 ± 2 °C).

Code	Cycle *	Vesicle Size, d_0.5_ (µm)				Distribution Uniformity			
		Day 1	Day 7	Day 30	Day 180	Day 1	Day 7	Day 30	Day 180
T13	1	0.195	0.196	0.194	0.193	0.110	0.421	0.099	0.089
T14	3	0.194	0.194	0.194	0.193	0.102	0.320	0.099	0.093
T15	5	0.195	0.184	0.193	0.193	0.100	0.168	0.098	0.092
**T16**	7	0.195	0.194	0.193	0.195	0.107	0.098	0.097	0.107
T21	1	0.086	0.093	0.093	0.093	0.089	0.256	0.259	0.255
T22	3	0.088	0.092	0.093	0.091	0.091	0.234	0.245	0.159
T23	5	0.087	0.090	0.091	0.091	0.080	0.308	0.235	0.116
**T24**	7	0.086	0.092	0.093	0.091	0.077	0.271	0.236	0.146

* Number of passes through the high-pressure homogenizer interaction chamber (Microfluidics M110P); d_0.5_, volume-weighted median diameter (laser diffraction).

**Table 3:** The pH, conductivity, density, and absolute zeta potential (ζ, mV) of resveratrol-loaded and unloaded ethosomes. Zeta potentials of phospholipid ethosomes are negative in sign; absolute values are shown in the table.

Code	pH	Conductivity (µS/cm)	Density (g/cm^3^)	ζ Day 1	ζ Day 7	ζ Day 14	ζ Day 30
T13	6.540	44.4	0.951	38.7	37.5	29.0	29.3
T14	6.508	44.5	0.961	36.0	34.2	24.8	33.1
T15	6.518	44.5	0.961	39.3	29.4	24.6	25.6
T16	6.500	44.6	0.949	41.2	33.9	28.5	25.8
T21	6.910	44.7	0.945	64.1	30.6	43.4	39.6
T22	6.721	53.2	0.948	76.7	50.6	52.4	54.4
T23	6.687	55.0	0.952	66.7	30.4	51.0	52.9
T24	6.653	56.8	0.956	67.2	43.7	40.7	55.6

**Table 4:** Encapsulated and unencapsulated trans-resveratrol in the optimized ethosome formulation (T16) on day 1 and after 180 days.

Code	% Unencapsulated (Day 1)	% Unencapsulated (Day 180)	% Encapsulated (Day 1)	% Encapsulated (Day 180)
T16 (resveratrol)	4.51	15.79	95.49	84.21

**Table 5:** Mean aspect ratio and image-based size dispersion (PDI) from cryo-SEM morphometric analysis.

Code	Mean Aspect Ratio *	Standard Deviation	PDI *
T16 (resveratrol)	1.43	0.28	0.038
T24 (unloaded)	1.37	0.22	0.026

* Interpreted as morphological size dispersion parameters, distinct from hydrodynamic PDI. PDI, polydispersity index; SD, standard deviation.

**Table 6:** Uniform cryo-SEM morphometric comparison of unloaded (T24) and resveratrol-loaded (T16) ethosomes.

Parameter	Unloaded (T24; BF0C1B1000)	Resveratrol-Loaded (T16; BF1C5B1000)
Objects analyzed, *n*	30	22
Projected area, mean ± SD (µm^2^)	1.74 ± 0.56	3.15 ± 1.26
Projected area, range (µm^2^)	0.82–2.83	0.54–6.02
Equivalent circular diameter, mean ± SD (µm)	1.47 ± 0.24	1.96 ± 0.42
Equivalent circular diameter, range (µm)	1.02–1.90	0.83–2.77
Mean aspect ratio ± SD	1.37 ± 0.22	1.43 ± 0.28
Image-based size dispersion (PDI)	0.026	0.038

Equivalent circular diameter computed from the projected area as d = 2√(area/π); aspect ratio and object counts are from the CfAM (centre for Advanced Microscopy) image analysis (University of Reading report). Per vesicle values and the indexed annotated micrographs are provided as source data in the Supplementary Materials.

## Data Availability

This study is part a Master of Science Thesis prepared by Hakan Sevinç under the supervision of Yasemin Yağan Uzuner. Some of the additional data is given in the Supplementary Materials. Any further information can be requested from the corresponding author.

## Supplementary Materials

The following supporting information can be downloaded at: https://www.mdpi.com/article/10.3390/pharmaceutics18101237/s1. Validation of the trans-resveratrol HPLC assay: Figure S1 (calibration curve/linearity); Table S1 (accuracy); Table S2 (intra-assay repeatability); Table S3 (repeatability at 220 µg·mL^−1^); Table S4 (robustness); and supporting sections on the limit of detection (LOD), limit of quantitation (LOQ), specificity, and solubility, copy of the graphs and tables embedded in the manuscript (the original of the device out-puts like FTIR spectrum, HPLC chromatograms are in picture form, the origin HaCaT Cell-line (HaCaT Cells 300493; Cytion GmbH, Heidelberg, Germany) Technical Data Sheets and Quality Control reports, and Cryo-SEM reports (Centre for Advanced Microscopy, University of Reading, Reading; UK). These micrographs represent unloaded ethosomes T24 (in the report, they are donated as (BF0C1B1000) and resveratrol-loaded ethosome T16, (BF1C5B1000). All these micrographs are supplied at a minimum of 1000 pixels in width/height (≥300 dpi) in the format exported by the microscope. In our study No electrophoretic blots or gels were used.
