# Supplementary material for: Ethosomal Nanocarriers for Trans-Resveratrol Delivery: Formulation, Physicochemical Characterization, Stability, and In Vitro Release Performance
Source: Pharmaceutics. 2026 Sep 29;18(10):1237. doi: 10.3390/pharmaceutics18101237 (PMC13708604; doi:10.3390/pharmaceutics18101237)
Supplement: Supplementary file 1 [file pharmaceutics-18-01237-s001.zip › pharmaceutics-4550926-supplementary.pdf]

Supplementary Information

Ethosomal Nanocarriers for Trans-Resveratrol Delivery: Formulation, Physicochemical Characterization, Stability, and In Vitro Release Performance

Yasemin Yağan Uzuner and Hakan Sevinç

The full HPLC method validation report (Supplementary Sections S1–S9: linearity, accuracy, precision, LOD, LOQ, robustness, and specificity), together with the trans-resveratrol solubility data (Figure S1; Tables S1–S4); the Certificate of Analysis of the trans-resveratrol raw material; the technical data sheet of the HaCaT keratinocyte cell line; and the full cryo-SEM report of the University of Reading (UK).

1. HPLC Method Validation and Solubility (Supplementary Sections S1–S9)

Validation of the trans-resveratrol HPLC assay (supporting Section 3.1.2). The assay method, instrumentation, and validation approach are described in Section 2.2 of the main text; the full linearity, accuracy, precision, LOD/LOQ, robustness, specificity, and solubility data are given below. Throughout, "the HPLC method" refers to the procedure in Section 2.2.

S1. Linearity

Trans-resveratrol solutions were prepared in ethanol at six concentrations and assayed by the HPLC method (Section 2.2). Over the concentration range used, the calibration curve was linear, with a regression equation $y = 1\times10^8x + 430677$ and a squared correlation coefficient $R^2 = 0.9994$ (Figure S1); the general acceptance criterion for linearity ($R^2 \geq 0.998$) was therefore met. The limit of detection (LOD) and limit of quantitation (LOQ) are reported in Sections S5 and S6.

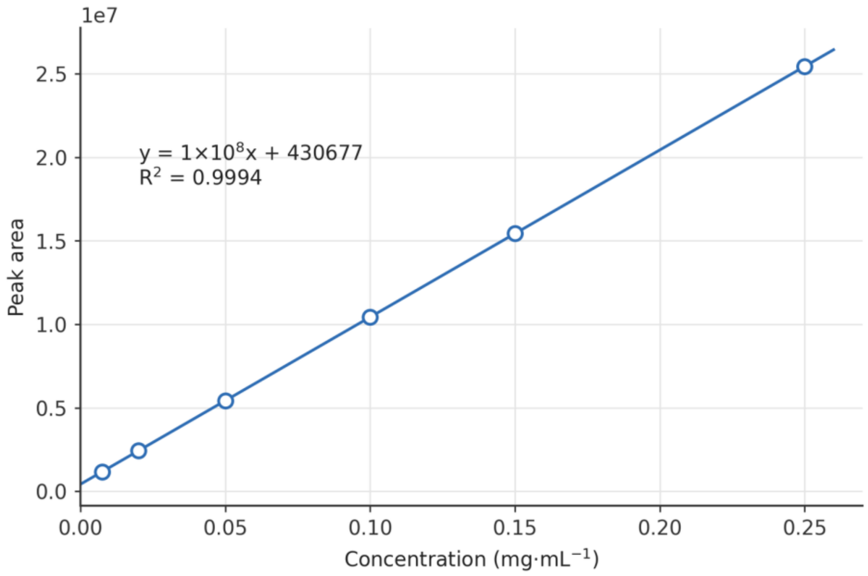

Figure S1. Calibration curve of trans-resveratrol obtained by the HPLC method (306 nm).

S2. Accuracy

Samples of trans-resveratrol were prepared in ethanol at 21, 50, and 100 μg·mL⁻¹ and measured five times by HPLC at 306 nm; the corresponding concentrations were calculated from the calibration equation (Table S1). The mean recoveries were within 102.6–104.3%, inside the accepted 90–110% range, confirming method accuracy.

Concentration	Mean recovery (%)	SD	CV
21 μg·mL⁻¹	102.80	0.12	0.001
50 μg·mL⁻¹	103.21	0.10	0.001

100 μg·mL⁻¹	104.22	0.06	0.001

Table S1. Accuracy of the trans-resveratrol assay (mean recovery, n = 5).

S3. Precision—Repeatability 1

Trans-resveratrol standards at 10, 20, and 110 μg·mL^{-1} were measured three times consecutively by HPLC at 306 nm (Table S2). The coefficients of variation were below 2%, indicating that the method is repeatable.

Concentration	Mean peak area	SD	CV
10 μg·mL^{-1}	1,306,583	2,700	0.002
20 μg·mL^{-1}	2,589,209	1,802	0.001
110 μg·mL^{-1}	11,099,561	214,206	0.019

Table S2. Intra-assay repeatability of the trans-resveratrol HPLC method (n = 3).

S4. Precision—Repeatability 2

Six samples prepared at 220 μg·mL^{-1} from the same stock (per Section 2.2) were measured by HPLC at 306 nm (Table S3). The coefficient of variation was below 2%, indicating that the method is reproducible.

Concentration	Mean peak area	SD	CV
220 μg·mL^{-1}	22,224,902	133,300	0.0060

Table S3. Repeatability of the trans-resveratrol HPLC method at 220 μg·mL^{-1} (n = 6).

S5. Limit of Detection (LOD)

The lowest concentration at which trans-resveratrol could be determined with acceptable accuracy and repeatability was calculated as 4.8 μg·mL^{-1}.

S6. Limit of Quantitation (LOQ)

The lowest concentration at which trans-resveratrol could be reliably quantified was calculated as 14.5 μg·mL^{-1}.

S7. Robustness

To assess the stability of trans-resveratrol in the dissolution medium, a 200 μg·mL^{-1} sample was measured twice within 24 h by HPLC (Table S4). No significant change was observed in the peak area (0.83% difference).

Concentration	1st measurement (area)	2nd measurement (area)	% Difference
200 μg·mL^{-1}	21,787,250	21,968,899	0.83

Table S4. Robustness of the trans-resveratrol HPLC method over 24 h.

S8. Specificity

The HPLC absorbance of a resveratrol-free (unloaded) ethosome formulation was measured as an unloaded reading. No

peaks coincided with the trans-resveratrol peak, confirming that the method is specific for trans-resveratrol; the representative chromatogram is shown as Figure 2 in the main text (retention time ~15.2 min).

S9. Solubility

The solubility of trans-resveratrol was ~65 $mg \cdot mL^{-1}$ in ethanol, ~100 $\mu g \cdot mL^{-1}$ in PBS (pH 7.2), and ~500 $\mu g \cdot mL^{-1}$ in PBS containing 2% polysorbate 20. These values informed the ethanol-based formulation and the surfactant-containing receptor medium used for the release studies (Sections 3.1.2 and 3.6.2).

2. Certificate of Analysis—Trans-Resveratrol (Raw Material)

The supplier's Certificate of Analysis for the trans-resveratrol raw material used in this study (≥98% trans-resveratrol, grape skin extract; Organic Herb Inc., Changsha, China) is provided as a separate file in this Supplementary package.

3. HaCaT Keratinocyte Cell Line—Technical Data Sheet

The technical data sheet and quality control report of the HaCaT keratinocyte cell line (HaCaT Cells 300493; Cytion GmbH, Heidelberg, Germany) used for the cytotoxicity assay are provided as a separate file in this Supplementary package.

4. Full Cryo-SEM Report—Centre for Advanced Microscopy, University of Reading (UK)

The complete cryo-scanning electron microscopy report (Centre for Advanced Microscopy, University of Reading, UK) is provided as a separate file in this Supplementary package. In that report the unloaded ethosomes (T24) are sample code BF0C1B1000 and the resveratrol-loaded ethosomes (T16) are BF1C5B1000; the panels used in Figure 6 of the manuscript are taken from these originals, and the per vesicle morphometry summarized in Table 6 was measured from them.
